# Brain-computer-interface-based intervention re-normalizes brain functional network topology in children with attention deficit/hyperactivity disorder

**DOI:** 10.1038/s41398-018-0213-8

**Published:** 2018-08-10

**Authors:** Xing Qian, Beatrice Rui Yi Loo, Francisco Xavier Castellanos, Siwei Liu, Hui Li Koh, Xue Wei Wendy Poh, Ranga Krishnan, Daniel Fung, Michael WL Chee, Cuntai Guan, Tih-Shih Lee, Choon Guan Lim, Juan Zhou

**Affiliations:** 1 Center for Cognitive Neuroscience, Neuroscience & Behavioral Disorders Program, Duke-National University of Singapore Medical School, Singapore, Singapore; 2 NYU Child Study Center, NYU Langone Medical Center, New York City, NY USA; 3 Department of Child and Adolescent Psychiatry, Institute of Mental Health, Singapore, Singapore; 4 School of Computer Science and Engineering, Nanyang Technological University, Singapore, Singapore; 5 Clinical Imaging Research Centre, the Agency for Science, Technology and Research, National University of Singapore, Singapore, Singapore

## Abstract

A brain-computer-interface (BCI)-based attention training game system has shown promise for treating attention deficit/hyperactivity disorder (ADHD) children with inattentive symptoms. However, little is known about brain network organizational changes underlying behavior improvement following BCI-based training. To cover this gap, we aimed to examine the topological alterations of large-scale brain functional networks induced by the 8-week BCI-based attention intervention in ADHD boys using resting-state functional magnetic resonance imaging method. Compared to the non-intervention (ADHD-NI) group, the intervention group (ADHD-I) showed greater reduction of inattention symptoms accompanied with differential brain network reorganizations after training. Specifically, the ADHD-NI group had increased functional connectivity (FC) within the salience/ventral attention network (SVN) and increased FC between task-positive networks (including the SVN, dorsal attention (DAN), somatomotor, and executive control network) and subcortical regions; in contrast ADHD-I group did not have this pattern. In parallel, ADHD-I group had reduced degree centrality and clustering coefficient as well as increased closeness in task-positive and the default mode networks (prefrontal regions) after the training. More importantly, these reduced local functional processing mainly in the SVN were associated with less inattentive/internalizing problems after 8-week BCI-based intervention across ADHD patients. Our findings suggest that the BCI-based attention training facilitates behavioral improvement in ADHD children by reorganizing brain functional network from more regular to more random configurations, particularly renormalizing salience network processing. Future long-term longitudinal neuroimaging studies are needed to develop the BCI-based intervention approach to promote brain maturation in ADHD.

## Introduction

Attention deficit/hyperactivity disorder (ADHD) is one of the most commonly diagnosed neuropsychiatric disorders of childhood affecting 3–10% of children^1^. Inattention is the common presentation of ADHD, representing approximately 38–57% of all ADHD cases in the community^2^. Children with inattention symptoms usually present with passive, lethargic attention problems or a deficit of sustained attention, such as procrastination, hesitation, and forgetfulness^2^. Clinically significant inattention and other functional impairment greatly affect their academic performance and social interaction, resulting in increased pressure and burden on their families and society^3^. Nevertheless, the etiological bases and neural substrates of ADHD are far from being fully understood and ADHD can be difficult to treat^4,5^.

The most common treatment for ADHD is pharmacotherapy. Medications used to treat ADHD such as methylphenidate, amphetamine, and atomoxetine indicate a dopamine/norepinephrine deficit as the neurochemical basis of ADHD, but the etiology is more complex. Moreover, these agents have poor adverse effect profiles and a multitude of drug interactions^4^. Therefore, despite the potential benefit of drug therapy for ADHD in children, medication must be dispensed with caution. Other standard therapy includes psychosocial or behavioral treatment, which may improve the social interaction problems, but have unknown efficacy for inattention problems^2,6^. Recently, electroencephalography (EEG)-based neurofeedback systems have been developed as an alternative modality for attention training and shown the effectiveness of recovery of function^7,8^. A few studies hypothesized that brain-computer-interface (BCI)-based neurofeedback system using specific EEG signals could induce neuroplastic changes in nervous systems^9,10^. Following this hypothesis, a BCI-based attention training game system was designed for treating ADHD children with significant inattentive symptoms^11^. The proposed system requires children to modulate their brain activity in attention training games, in which feedback represents the measured concentration level. It entails the value of maintaining player interest and utilizing the virtual situation to maximize the transferability to real-life contexts. Following the BCI-based training, parent-rated inattentive and hyperactive-impulsive symptoms on the ADHD Rating Scale (ADHD-RS) showed significant improvement in children with ADHD, exhibiting perspectives to be a potential new treatment for ADHD. The treatment effects of such neurofeedback-based training in children with ADHD were thought to relate to the successful regulation of brain activity and the ability of the brain to change and adapt, known as brain plasticity^8,9^.

Accumulating evidence suggests that non-invasive neuroimaging methods can provide in vivo insights on brain plasticity at the macro level of large-scale functional networks, uncovering the mechanisms underlying both loss and recovery of function^12^. Several intrinsic connectivity networks (ICNs) playing distinct functional roles have been consistently identified in healthy individuals using resting-state functional magnetic resonance imaging (RS-fMRI), which measures correlated low-frequency blood-oxygenation-level-dependent (BOLD) signal fluctuations between brain regions under resting or task-free condition^13–16^. This discovery has also opened new avenues for investigating the developing brain. Among these ICNs, large-scale cognitive networks undergo significant developmental reconfigurations and maturations to support more flexible cognitive control processes in adulthood. For example, the salience network is responsible for orienting attention to salient stimuli and internal events and the default mode network (DMN) is associated with self-referential mental activity. Deficits in these cognitive networks play a significant role in many psychiatric and neurodevelopmental disorders, including ADHD^17,18^.

Recent evidence suggest altered intrinsic organization of brain networks is implicated in the dysfunction of the ADHD disorder^19^, which displayed hyper-connectivity within the DMN and ventral attention network and between ventral attention and dorsal attention networks (DAN)^20,21^. Furthermore, graph theory has been recently applied in functional connectomics analysis to elucidate the complex network organization at the regional and system level^22^. Graph theoretical analysis found that children with ADHD showed abnormal small-world architecture characterized by higher local clustering (high local efficiency) combined with a tendency of lower global efficiency as compared to healthy controls, exhibiting a shift toward the configuration of regular networks^23^. A regular network has a high local efficiency (associated with local or segregated processing) and low global efficiency (associated with distributed or integrated processing) while a random network has a low local efficiency and high global efficiency^24^. This abnormality in children with ADHD pointed to a developmental lag of whole-brain functional networks in them. Previous studies have demonstrated that the maturation of the healthy developmental human brain follows a “local to distributed” principle, specifically a reduction in local efficiency and increase in global efficiency, suggesting a shift of topological organization toward more random configurations^25,26^.

Although the BCI-based training has shown promise in behavioral improvement in children with ADHD, whether and how these behavioral improvements are supported by functional network changes facilitating brain maturation following the BCI-based treatment in children with ADHD remain largely unknown. To address this gap, we aimed to examine whether and how the large-scale brain networks reorganize after the 8-week BCI-based attention treatment in children with ADHD using RS-fMRI imaging. Given previous findings on more regular configurations and higher intra- and inter-network connectivity in ADHD, we hypothesized that after the intervention, the brain functional network of childhood ADHD would have lower intra- and inter-network connectivity especially in task-positive networks and move from more regular configurations toward more random configurations accompanying their behavioral improvement. We also sought to test whether changes in functional connectivity and topological measures in ADHD were associated with changes in clinical symptoms.

## Materials and methods

### Participants

We studied 66 boys with ADHD, either combined or inattentive subtypes, recruited from the Child Guidance Clinic, Institute of Mental Health, Singapore. The diagnoses were made by child psychiatrists according to the Diagnostic and Statistical Manual-Fourth Edition (DSM-IV) for ADHD^27^. Parents were also interviewed using the Diagnostic Interview Schedule for Children based on DSM-IV. ADHD participants on medicine were only allowed to participate after at least 1 month of washout. Exclusion criteria included history of epileptic seizures, mental retardation, and an intelligence quotient of <70, which was measured using Kaufman Brief Intelligence Test, Second Edition. Written informed consent from parents and assent consent from children were obtained and ethical approval was governed by National Healthcare Group IRB, Singapore. The ADHD participants were randomly divided into two groups as part of another larger behavioral clinical trial and their involvement in this subcohort of neuroimaging study was on a purely voluntary basis. As a result, we studied 8-week BCI-based intervention group (ADHD-I, *N* = 44) and non-intervention group (ADHD-NI, *N* = 22). As there was no prior neuroimaging data using this novel BCI-based intervention, based on the effect size reported in one prior fMRI study of neurofeedback therapy showed significant changes in the brain with a sample size of 15 in treatment group, we estimate 30 per group would be adequate after taking into account the 20% losses to follow-up^28^. MRI scans and clinical assessments at baseline and follow-up were obtained for all participants. The researchers were blinded to the group allocation during the MRI scans and clinical assessments. Of the 66 participants, 15 subjects had incomplete MRI data due to various reasons, including dropping out and scan tolerance. Therefore, 51 participants (ADHD-I, *N* = 33; ADHD-NI, *N* = 18) had both T1 and RS-fMRI imaging data for both scan sessions. After careful quality control, 18 subjects from ADHD-I group and 11 subjects from ADHD-NI group have good structural and functional MRI data at both time points. The two groups were matched in age, handedness, ethnicity, and motion parameters (i.e., number of RS-fMRI volumes left for analysis after motion scrubbing) (Table 1).

**Table 1:** Demographic and imaging information of the participants

	ADHD-I (*N* = 18)		ADHD-NI (*N* = 11)		*p*-Value
	Time point 1	Time point 2	Time point 1	Time point 2	
Age, mean (SD), years	9.00 (1.50)		9.45 (1.29)		0.412
Gender	All males		All males		–
Handedness	All right		All right		–
Ethnicity	All Chinese		10 Chinese, 1 Indian		0.193
Scanner type	5 Tim Trio, 13 Prisma		9 Tim Trio, 2 Prisma		0.005*
Number of volumes left after motion scrubbing, mean (SD)	208.389 (21.136)	203.889 (32.881)	193.727 (39.664)	208.636 (27.496)	0.595
Mean absolute motion displacement (mm)	1.037 (0.975)	0.870 (0.571)	1.339 (1.024)	1.384 (0.820)	0.327
Max. absolute motion displacement (mm)	2.321 (1.370)	2.227 (1.396)	3.021 (1.777)	2.945 (1.412)	0.367
ADHD-RS inattention score	16.278 (4.254)	13.167(4.077)	18.909 (5.186)	17.273(5.764)	0.148^△^: 0.038^+^
CBCL internalizing problems	7.889 (5.086)	5.389 (4.175)	12.364 (9.553)	10.546 (7.841)	0.110^△^: 0.441^+^

*N* number of subjects. “*” indicated there was significant difference with *p*-value < 0.05. “^△^” indicated the test was performed between the two groups at the first time point. “+” represents the interaction effect between group and time

### BCI-based intervention procedure

**Fig. 1:**
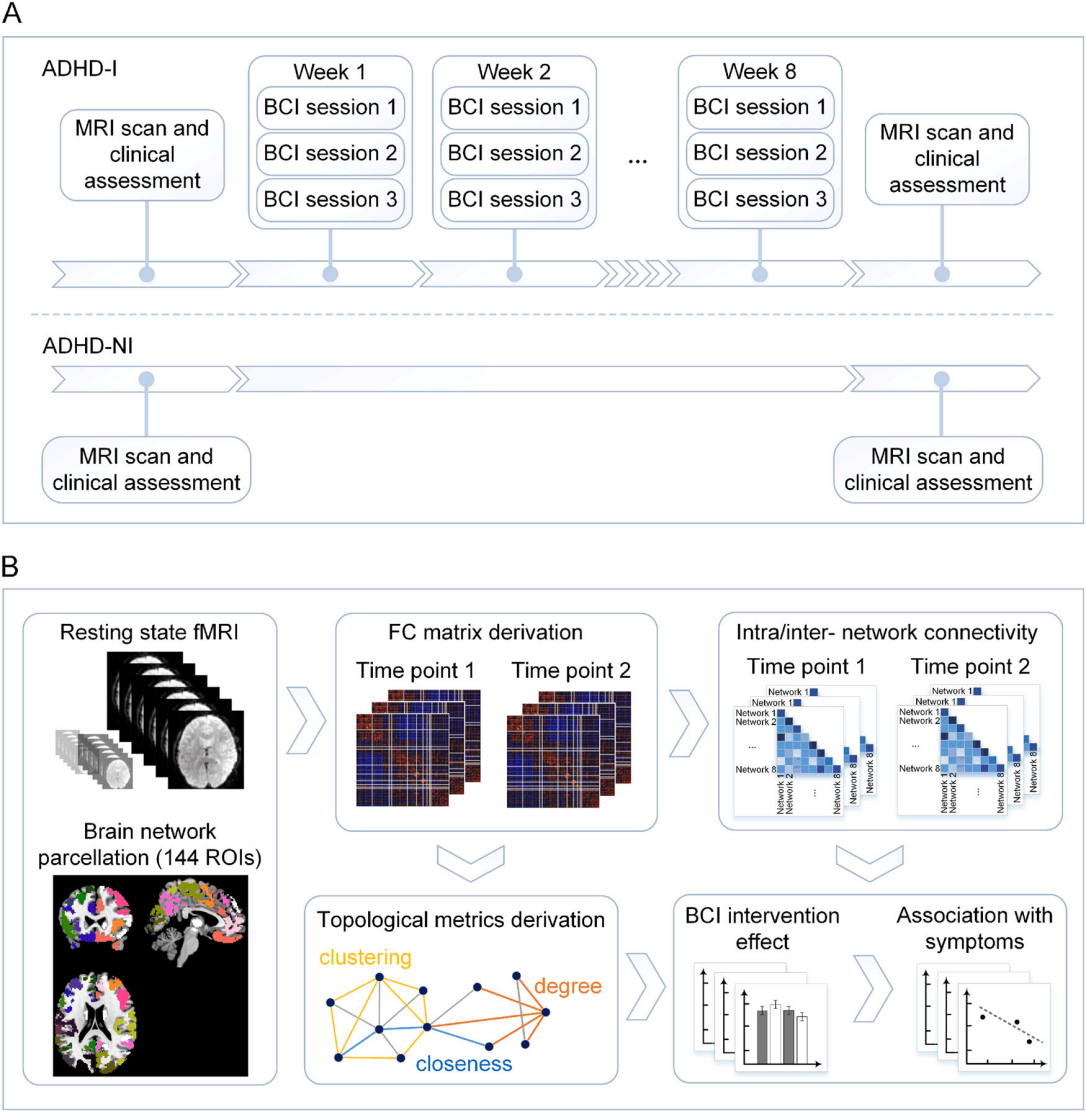
Study design schematic diagram. **a** Participants were randomly divided into two groups: intervention group (ADHD-I) and non-intervention group (ADHD-NI). All participants underwent resting-state functional magnetic resonance imaging (RS-fMRI) and neuropsychological assessments at baseline and follow-ups. Between the two visits, participants in ADHD-I group underwent a brain-computer-interface (BCI)-based attention game training (three sessions per week for 8 weeks). **b** The functional connectivity (FC) matrix among 141 regions of interest (ROIs) covering the whole brain was derived for each participant at each time point. Intra- and inter-network FC measures were calculated. The FC matrix was then thresholded to a sparse weighted network to derive network topological measures. These FC metrics were then used to examine the effect of the BCI-based intervention on brain networks and brain-behavioral associations

The ADHD-I participants underwent three BCI-based training sessions per week for 8 weeks (Fig. 1). For each training session, individuals would complete 30 min BCI-based training, including the breaks. The BCI-based attention training game system consisted of a headband with mounted dry EEG sensors (manufactured by Zeo, Inc., Boston, Massachusetts, USA) that transmitted EEG readings to the computer through Bluetooth-enabled protocol (see details in our previous work^11^). Briefly, the headband was worn around the forehead, with a grounding reference electrode clipped to the earlobe. Two dry EEG electrode sensors were positioned at the frontal sites FP1 and FP2. The advanced signal processing techniques based on machine learning algorithm pick up useful information about attentional activities from the recorded frontal EEG signals and then send the feedback using the computerized three-dimensional (3D) graphic game (CogoLand) presented on the screen^11^. In the game, each participant controlled an avatar to complete a task, for example, making the avatar run around an island in the shortest time possible. The avatar ran faster if participants were more attentive. A short break was allowed between attempts.

### Neuropsychological assessments

Neuropsychological assessments, including the ADHD-RS^29^ and the Child Behavior Checklist (CBCL)^30^ were administered on children with ADHD at baseline and after BCI-based training. ADHD-RS is an essential part of the full assessment process for ADHD while the CBCL is a parent-rated questionnaire designed to obtain descriptions of a child’s competencies and behavioral/emotional problems, which provides both empirical-based symptoms and dimensional constructs for psychopathology. We expected the attention would be significantly improved in the participants with ADHD through the BCI-based treatment, hence the ADHD-RS clinician inattentive score were used as the primary outcome. As ADHD often co-occurs with internalizing disorders as previously reported^31^, the CBCL internalizing problems scale, which comprises problems that are mainly within the self, reflecting anxiety disorder, and social phobia, was also used as secondary outcomes. The ADHD-NI and ADHD-I groups (after imaging quality control) had comparable ADHD-RS clinician inattentive scores and CBCL internalizing problem scores at baseline (Table 1).

### Image acquisition

All functional and structural MRI images were collected at the Center for Cognitive Neuroscience, Duke-National University of Singapore Medical School using a 12-channel head coil on a 3-T Tim Trio or a 20-channel head coil on a 3-T Prisma scanner (Siemens, Germany) due to unavoidable system upgrade. The same imaging parameters were used for both scanners for maximum consistency. Moreover, during the scanner upgrade, we conducted a test-retest study to ensure comparability of T1 and fMRI data between the old and new scanners (see Supplementary Methods and Results). The RS-fMRI data using T2*-weighted echo planar images (repetition time = 2000 ms, echo time = 30 ms, flip angle = 90°, field of view = 192 × 192 mm^2^, voxel size = 3.0 mm isotropic, slice thickness = 3 mm, no gap, 36 axial slices, interleaved collection) were collected while the subjects were asked to relax and stare at a cross centered on a screen. The RS-fMRI data collection (8 min 12 s altogether; 246 volumes) was broken up into two consecutive short runs to minimize motion artifacts; the duration for each of the two runs were 4 min 6 s each. We concatenated the two runs of RS-fMRI data for further processing. An eye tracker was used to ensure that the children stayed awake for the entire RS-fMRI scan. The high-resolution structural T1-weighted magnetization prepared rapid gradient echo images (repetition time = 2300 ms, echo time = 2.98 ms, inversion time = 900 ms, flip angle = 90°, field of view = 256 × 256 mm^2^, voxel size = 1.0 mm isotropic) were collected for atlas registration of the RS-fMRI images. To minimize the influence of scanner difference, we included the scanner type as a covariate in all statistical analysis.

### Image preprocessing

RS-fMRI images and structural MRI images were both preprocessed using a standard pipeline based on the FMRIB’s Software Library (FSL, www.fmrib.ox.ac.uk/fsl)^32^ and the Analysis of Functional NeuroImages software program^33^ following our previous work^34,35^. The structural image preprocessing included: (1) image noise reduction; (2) skull stripping; (3) linear and nonlinear registration to the Montreal Neurological Institute (MNI) 152 standard space; and (4) segmentation of the brain into gray matter, white matter, and cerebrospinal fluid (CSF) compartments. Preprocessing steps for the RS-fMRI data included (1) discarding the first five volumes and interleaved slice-timing correction, (2) motion correction using first functional image with skull, (3) skull stripping, (4) despiking and grandmean scaling, (5) spatial smoothing using a 6 mm full-width half-maximum Gaussian kernel to improve signal-to-noise ratio and to reduce inter-subject variability, (6) temporal band-pass filtering (0.009–0.1 Hz) and detrending (first and second order), (7) structural MRI co-registration using Boundary-based Registration, and nonlinear registration (FNIRT) to the MNI 152 stereotactic standard space of 2 mm isotropic resolution, and (8) nuisance signals reduction by regressing out signals estimated from CSF, white matter, and six motion parameters. We did not regress out the global signal because the global signal may contain neural information^36^. Registration and normalization quality was visually inspected. Subsequently, we performed motion scrubbing to minimize spurious functional connectivity in brain networks. Frame displacement (FD) and the rate of change of BOLD signal across the entire brain (DVARS) at each frame were calculated^37^ and frames with FD larger than 0.8 and DVARS larger than 0.05 were removed.

### Functional connectivity matrices

We derived the individual whole-brain functional connectivity matrix based on mean time series extracted from a set of 144 regions of interest (ROIs) defined by a previous data-driven functional parcellation scheme^38,39^. The 144 ROIs can be grouped into seven ICNs (the salience/ventral attention network (SVN), the DAN, the DMN, the executive control network (ECN), the somatomotor network (SMN), the visual network, and the limbic network) and 30 subcortical regions. Due to the lack of coverage in certain brain scans, 141 ROIs were used for network construction. At the individual level, we calculated the Pearson’s correlation between the time series of each pair of ROIs and then Fisher’s *r*-to-*z* transformed it into the FC *z*-score matrix. We then summarized the intra-network FC and inter-network FC between seven major ICNs and subcortical regions for statistical analyses.

### Graph theoretical measure derivation

To characterize the brain network topology, we derived graph theoretical measures from individual-level weighted FC matrices for both time points. In graph theory, each ROI stands for a node, and the FC of each pair of the nodes is defined as an edge. We focused on degree centrality, clustering coefficient, and closeness at the nodal level and efficiency, clustering coefficient, and small-worldness at global level using in-house Matlab scripts based on Brain Connectivity Toolbox^22^ following our previous work^34^, to evaluate the network integration and segregation features (see Supplementary Methods). Small-world topology is one of the fundamental characteristics of brain networks^40^, i.e., the mean shortest path between nodes increases sufficiently slowly as a function of the number of nodes in the network. It is defined as the ratio of the normalized clustering coefficient to the normalized shortest path length^41^. The global measures investigated were the global efficiency and clustering coefficient, to quantify the overall extent to which the networks were integrated and segregated respectively. The nodal measures assessed, namely the nodal degree, closeness, and clustering coefficient, were computed for each of the 141 nodes separately. The degree is the most commonly used metric for node centrality, which provides an evaluation of how the node is connected to the other nodes in the network. The nodal closeness and clustering coefficient quantified the extent to which each particular region was integrated within the network and segregated among its immediate neighbors, respectively^22^.

Only positive FC values were considered while negative values were set to zero. To make sure that results were not contingent upon the choice of a specific network density threshold, we derived the three topological measures from FC matrices across a variety of network density thresholds (15–35% with a step of 1%, see steps to determine the range in Supplementary Materials) and then took the integration across all thresholds for statistical analyses.

### Statistical analyses

To test whether ADHD-I group had behavioral improvement compared to ADHD-NI after the BCI-based training, two-way repeated analysis of variance (ANOVA) was performed on internalizing problems and inattention scores.

To examine the possible group (ADHD-I vs. ADHD-NI) and time (pre- and post-BCI training) interaction effect on brain functional connectivity, we performed two-way repeated ANOVA on intra-network and inter-network FC values using permutation testing (5000 permutations, reported at the alpha level of 0.05). The individual effects of age and scanner type were included as covariates for all the tests. Moreover, we repeated the same two-way repeated ANOVA on the global and nodal graph theoretical measures to test the possible BCI-related network topological changes.

We then sought to test if the identified brain functional connectivity changes were associated with changes in symptom severity across ADHD patients using Pearson’s correlation analysis. Consistently, the individual effects of age and scanner type were regressed out as covariates from the neuropsychological assessments.

### Code availability

Requests for code can be addressed to the corresponding author.

## Results

### BCI-based intervention improved attention in ADHD

**Fig. 2:**
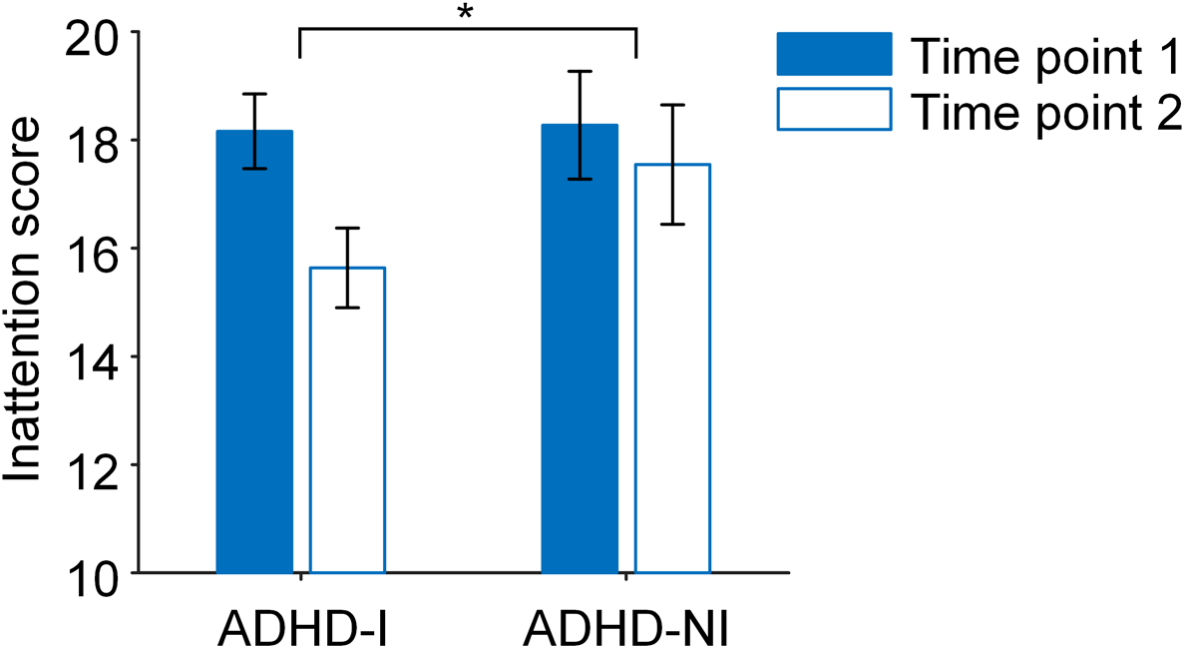
BCI-based intervention improved the attention in ADHD. The ADHD-I group had significantly greater reduction in the ADHD-RS clinician inattention scores compared to the ADHD-NI group (*p* = 0.038)

After 8-week BCI-based intervention, the ADHD-I group had significantly greater reduction in the ADHD-RS clinician inattention scores compared to the ADHD-NI group (*p* = 0.038, Fig. 2). The reduction of CBCL internalizing problems in ADHD-I group was slightly greater than that in ADHD-NI group, but not significant (*p* = 0.44).

### BCI-based intervention re-balanced task-positive functional networks

**Fig. 3:**
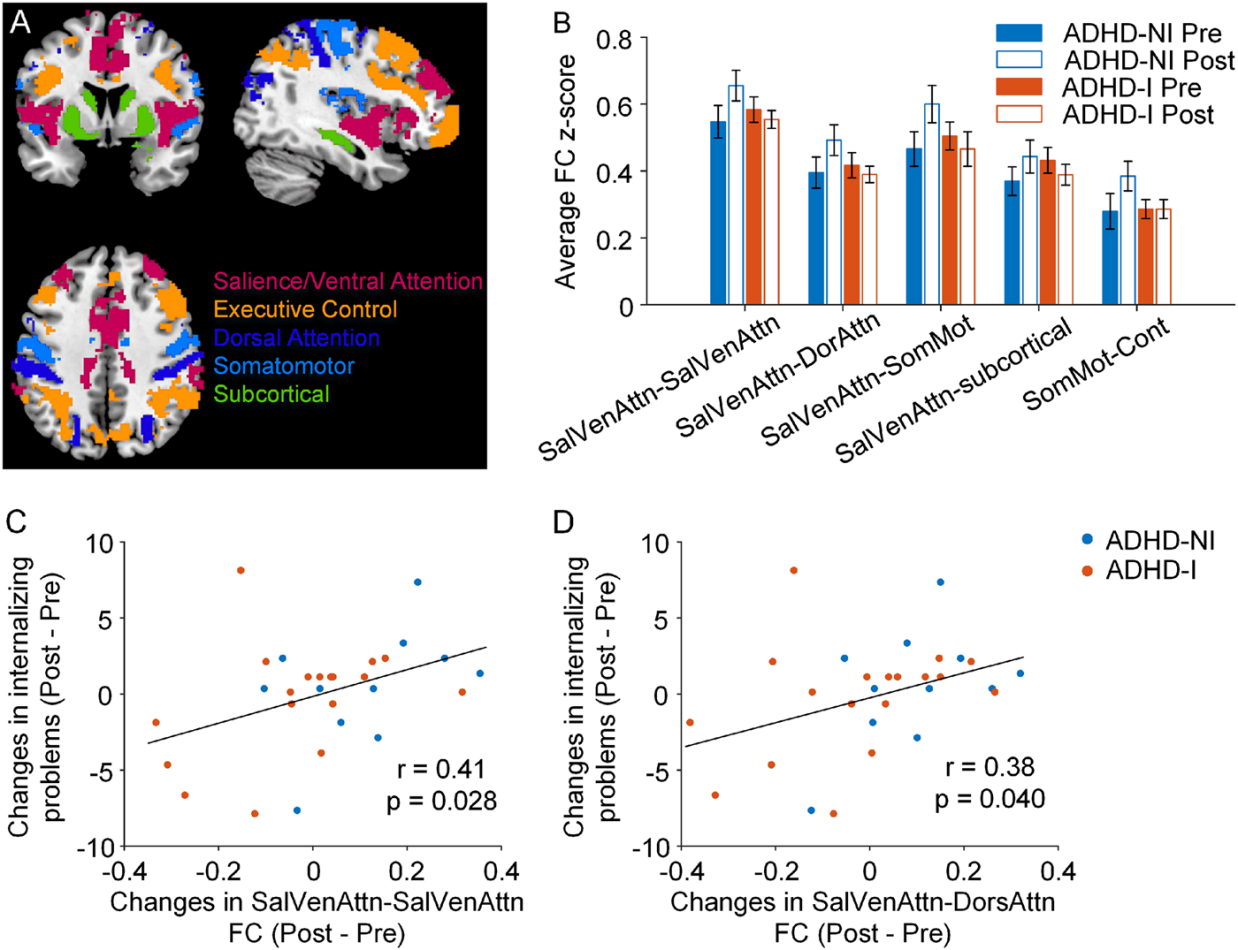
Changes in intra- and inter-network functional connectivity (FC) of the attentional networks related to behavioral improvement in ADHD after the BCI-based intervention. **a** Brain slices highlight the major intrinsic connectivity networks and subcortical regions^38^. **b** Intra- and inter-network FC showed significant group and time interaction effect (*p* < 0.05). Error bars represent standard errors. ADHD-NI group had increased FC within the salience/ventral attentional network (SVN) and between the SVN with dorsal attention (DAN) and other networks while ADHD-I did not exhibit this pattern. **c**, **d** FC changes of the intra-SVN and the inter-network between SVN and DAN by the BCI-based intervention were correlated with the behavior improvement of internalizing problems in ADHD children. SalVenAttn: salience/ventral attention network, DorAttn: dorsal attention network, SomMot: somatomotor network, Cont: executive control network

We found significant group and time interaction in the FC within the SVN (*p* = 0.019) and between the SVN and DAN (*p* = 0.035), SVN and SMN (*p* = 0.014), SVN and subcortical network (*p* = 0.050), and SMN and ECN (*p* = 0.049, Fig. 3b, Supplementary Table 2), which indicated a trend of ADHD-NI group having increased FC within the SVN and between the SVN with DAN and other networks over time while ADHD-I did not exhibit this pattern.

Among the FC measures showing significant time and group effect, we found that less increase of FC in the intra-SVN and the inter-network between SVN and DAN resulted in more behavior improvement of the internalizing problems in children with ADHD (*r* = 0.41, *p* = 0.028 and *r* = 0.38, *p* = 0.040, respectively; Fig. 3c, d).

### BCI-based intervention re-normalized brain network topology

Global efficiency and clustering coefficient did not show any significant effect of the BCI-based training over time (*p* > 0.05). In contrast, the small-worldness measure showed a significant time and group interaction (*p* = 0.045). After the BCI-based training, the small-worldness of the ADHD-I group remained almost the same while the small-worldness of the ADHD-NI group decreased significantly. Moreover, reduction of small-worldness was correlated with less behavioral improvement (CBCL internalizing problems) over time across all ADHD patients (*r* = −0.384, *p* = 0.040).

**Fig. 4:**
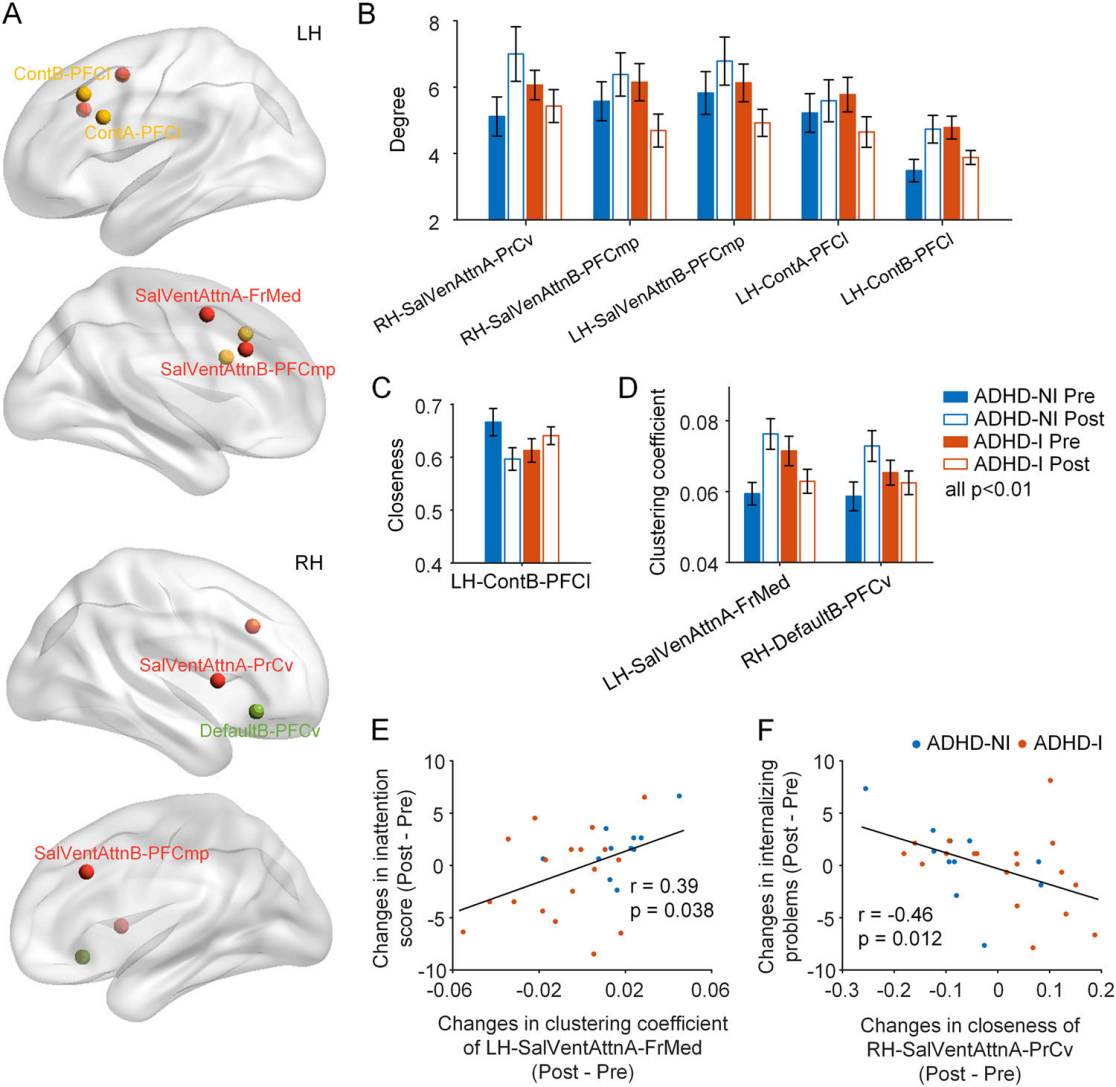
BCI-based intervention in ADHD is associated with brain network re-organization underlying behavioral improvement. **a** Nodes showing significant time and group interaction effect on nodal degree, clustering coefficient, or closeness are presented. Brain network topology exhibited significant group and time interaction in nodal degree (**b**), clustering coefficient (**c**), and closeness (**d**) (*p* < 0.05). Error bars represent standard errors. Changes of nodal graph metrics by the BCI-based intervention were correlated with the behavior improvement of internalizing problems and inattention in ADHD children (**e**, **f**). ContA/B executive control network A/B (A or B refers to the subnetworks), SalVenAttn: salience/ventral attention network, DorAttn: dorsal attention network, Default: default mode network, PrCv: precentral ventral frontal cortex, PFCmp: medial posterior prefrontal cortex, PFCl: lateral prefrontal cortex, SPL: superior parietal lobule, FrMed: medial frontal cortex, PFCv: ventral prefrontal cortex

For nodal measures, the nodal degree and clustering coefficient were reduced and the nodal closeness was increased after the BCI-based intervention, mainly in the SVN, ECN, and DMN (*p* < 0.01, Fig. 4b–d, Supplementary Table 3). To provide a more complete picture, supplementary Figure 1 presented all nodal measures at a lower threshold of *p* < 0.05. These brain network topological changes suggested reduced local functional processing in task-positive networks and the DMN (mainly prefrontal regions) after BCI-based training.

More importantly, among the nodal graph theoretical measures showing significant time and group effect, we found that less increase of the degree and the clustering coefficient of several DMN, SVN, and visual network nodes related to more behavior improvement of the inattention scores and the internalizing problems in children with ADHD. Similarly, less decrease of the closeness in three SVN nodes were associated with more behavior improvement of the internalizing problems in children with ADHD (*p* < 0.05, Fig. 4e, f and Supplementary Figure 2).

In addition, we analyzed the motion-related measures (FD and DVARS) in the two ADHD groups in terms of possible BCI-intervention effects. The in-scanner head motion parameters were comparable between the two groups and across the two time points, which did not reflect BCI-based intervention effect (see Supplementary Methods and Results).

## Discussion

We presented evidence for brain network topology reconfigurations by the BCI-based intervention in children with ADHD. Following the BCI-based training, FC was decreased within the SVN, and between task-positive networks and subcortical regions. In parallel, the nodal degree and clustering coefficient were reduced and the nodal closeness was increased in nodes mainly from task-positive networks, DMN, and visual network, suggesting reduced local functional processing after BCI-based training. Importantly, these functional network re-organization were correlated with the improvement of inattention symptom and internalizing problems in ADHD children. Our findings highlight the value of network-sensitive neuroimaging methods to uncover brain plasticity mechanism related to intervention efficacy in neurodevelopmental disorders such as ADHD.

### BCI-based intervention improved ADHD symptoms

This study utilized a BCI-based attention training program in the treatment of combined and inattentive subtypes of ADHD. The results showed that an 8-week intervention significantly improved inattentive symptoms of ADHD children based on ADHD-RS inattention subscale. There are several advantages the BCI-based attention training program can offer: it is non-pharmacological, much safer, and easy to learn and it can be done at home, the system represents a novel treatment modality for ADHD childhood, which not only has the potential for being used in combination with present evidence-based treatment but also uniquely in a nonclinical setting for its convenience.

### BCI-based intervention regularized the salience processing in ADHD

The changes of ADHD brain network by the BCI-based intervention we revealed majorly involved the SVN, which might bring some interesting aspects to note. The salience network has a central role in the detection of behaviorally relevant stimuli, integrating information, the coordination of neural resources, and the mediation of information flow between other networks involved in higher order cognition, such as default mode and ECN^42,43^. Recent evidence suggests that dysregulation of salience-processing systems can occur in many brain conditions, including neurodevelopmental disorders^44,45^. Emerging imaging evidence points to the dysfunctional coordination of the attention networks and DMN, controlled by salience network, contributing to attentional engagement, and disengagement in ADHD^46^. The attention systems, ventral attention network and DAN, have specialized roles as well as interaction: the DAN is involved in top-down voluntary allocation of goal-driven attention, whereas the ventral attention network is involved in the stimulus-driven attention^20^. Our data suggest that the BCI-based treatment had the SVN connectivity decreased, and the SVN and DAN more functionally segregated in ADHD children, and importantly, these two changes were significantly correlated with the behavior improvement, potentially indicating that the attention training games re-normalized the salience-processing system and the effective coordination between the two attention systems.

We also found reduced connectivity between SVN and subcortical and SMN following the BCI-based intervention in ADHD, indicating that the BCI-based intervention also altered the interaction between the salience network and these two important networks. From the pathophysiological basis, the general distractibility in ADHD has been attributed to a deficit in dopaminergic signaling in subcortical-cortical networks that regulate goal-directed behavior^47^, and there was some evidence for differential abnormalities in the basal ganglia^48^. The BCI-based intervention induced reduced connectivity between SVN and subcortical network might reflect the renormalization of subcortical network FC.

### BCI-based intervention facilitated brain maturation in ADHD

The human brain is functionally organized into hierarchical, modular structure, and the network modules become more segregated with age in neurotypical development, allowing for specialized processing to occur within densely interconnected groups of brain regions, which reduce interference among systems and facilitate cognitive performance^26,49^. Also, across development, the topological organization of multiple functional networks shifts from a local anatomical emphasis to a more “distributed” architecture, specifically a reduction in local efficiency and increase in global efficiency, suggesting a shift of topological organization toward more random configurations^25^. It has been proposed that ADHD involves a delayed or altered maturation of brain’s developing functional architecture as well as its structural features such as gray matter volume or cortical thickness^50^. The developmental delay in functional connectivity patterns in children with ADHD lead to a more regular configuration of networks, characterized by higher local efficiencies and a tendency of lower global efficiency^23^. In this study, we found decreased connectivity in inter-networks between the task-positive networks by the BCI-based intervention, indicating increased segregated processing in children of ADHD-I group. In parallel, we found the BCI-based intervention led to decreased nodal clustering coefficient indicating decreased local efficiency in children of ADHD-I group, which suggest a trend toward a more random topology configuration. In contrast, the ADHD-NI group did not have such pattern over time or had the rather opposite trajectory (Supplementary Tables 2 & 3). Importantly, some of the topological changes were correlated with the behavioral improvements of inattention and internalizing problems. We suspect that the BCI-based intervention may modify the brain developmental trajectory, possibly leaning toward the healthy pattern, and thus result in improved behavior in ADHD patients. Taken together, the study suggested that the BCI-based intervention to a degree avert the lag of brain maturation from the perspective of topological architecture of brain network.

### Limitations and future directions

The primary limitation of our study was the relatively small sample size after the removal of poor-quality data due to excessive motion. Second, physiological noise in the fMRI dataset could be further corrected by implementing advanced fMRI preprocessing techniques^51^. Furthermore, our study did not distinguish between the ADHD subtypes due to the limited sample size. Although both the inattentive and combined subtypes have predominant inattention problems, each subtype may have unique response of brain networks following the BCI-based treatment. It is however noted that the subtyping of ADHD has been removed in DSM-5. Further studies may need to take the clinical heterogeneity of ADHD into account. Further studies were needed to study ADHD patients with longer duration of BCI-based treatment and long-term follow-ups.

In conclusion, our study revealed the neural mechanism underlying behavioral improvement following a BCI-based training on children with ADHD. The BCI-based intervention can help re-normalize brain functional network topology among cognitive networks, which is associated with behavioral improvement and facilitate brain maturation in ADHD children. These findings underscore the potential value of BCI-based attention training game as an attractive treatment strategy for ADHD.

## Electronic supplementary material

supplemental material
