# Supplementary material for: Brain-computer-interface-based intervention re-normalizes brain functional network topology in children with attention deficit/hyperactivity disorder
Source: Transl Psychiatry. 2018 Aug 10;8:149. doi: 10.1038/s41398-018-0213-8 (PMC6086861; doi:10.1038/s41398-018-0213-8)
Supplement: Supplementary file 1 — supplemental material [file 41398_2018_213_MOESM1_ESM.docx]

**SUPPLEMENTARY METHODS**

**Graph theoretical analysis**

We first thresholded the subject-level functional connectivity (FC) matrix using a range of costs (i.e., the ratio of the total number of edges to the maximal number of possible edges) from 0.15 to 0.35 in 0.01 steps to ensure that 90% of the nodes were connected and that small-worldness was greater than 1.2 at the extremities (1). Nodal degree, closeness and clustering coefficient and global efficiency, clustering coefficient and small-wordness (2) were then derived from the individual FC matrix at each of the 20 cost thresholds within the range using in-house scripts based on the Brain Connectivity Toolbox (2). The mean graph theoretical metrics, i.e. calculated by integrating over the cost threshold range and then dividing by the cost range, were used for statistical examination of (ADHD-I versus ADHD-NI) and time (pre- and post- BCI training) interaction effect.

**Comparability of functional connectivity between the two scanners**

This study involved the use of two scanner types due to unavoidable system upgrade. During the scanner upgrade, we conducted a test-retest study to ensure comparability of T1 and fMRI data between the old and new scanners. We recruited 23 young adults (11 females, mean (SD) age of 28.70 (6.03) years). All the participants underwent one T1-weighted structural MRI scan and one 8-min task-free fMRI scan (eyes-open with fixation) using 12-channel head coil on 3-Tesla Tim Trio and 20-channel head coil on 3-Tesla Prisma scanner before and after system upgrade. The mean (SD) duration between scans is 94.48 (23.98) days. The motion parameters were comparable between the two time points (p > 0.05). RS-fMRI images and structural MRI images were both preprocessed using the same standard pipeline as described in methods. To derive the individual brain FC, we performed seed-based correlation analysis following our previous work (3) as well as the 141-ROI-based FC matrix analysis described in methods. For the seed-based approach, the DMN seed was selected from the peak foci of intrinsic connectivity networks reported in previous literature (right angular: MNI coordinates: 46, - 60, 22) (4). Pearson’s correlation was computed between each voxel’s spontaneous BOLD time series and the average time series from the 4-mm radius spherical seed for each preprocessed functional image. The Pearson’s correlation was converted to z-scores using Fisher’s r-to-z transformation.

The test-retest reliability of FC measures and BOLD signal variability in the 141 ROIs across the two time points were quantified following previous work as: a) intra-class correlation coefficient (ICC) across subjects (5); b) subject-wise concordance correlation coefficient (CCC) of spatial variation (6). Both approaches were performed on BOLD signal variability, seed-based FC measures, and 141-ROI-based FC matrices. Following our previous study (7), test-retest reliability was characterized as excellent (> 0.8), good (0.6 - 0.79), moderate (0.4 - 0.59), fair (0.2 - 0.39) or poor (< 0.2).

**Comparison of motion parameters of the two groups across two sessions**

In this study, there were 51 participants (ADHD-I, N=33; ADHD-NI, N=18) having imaging data available for both sessions. After careful quality control, 29 participants (ADHD-I, N=18; ADHD-NI, N=11) had good imaging data for both sessions. Therefore, we performed statistical analysis on the FD and DVARS on both the complete sample (33 ADHD-I and 18 ADHD-NI patients) and sub-sample after quality control (18 ADHD-I and 11 ADHD-NI patients). For each individual, we derived the FD and DVARS from preprocessed rs-fMRI data with and without motion scrubbing. To examine the time-group interaction effect, we performed two-way repeated ANOVA on the FD and DVARS across the two time points on the complete sample (ADHD-I, N=33; ADHD-NI, N=18) and sub-sample after QC (ADHD-I, N=18; ADHD-NI, N=11) respectively. We also performed the paired t-test on the FD and DVARS at baseline and post-intervention for each group.

**SUPPLEMENTARY RESULTS**

**Comparability of functional connectivity between the two scanners**

We have kept the same imaging parameters (T1 and fMRI) before and after the system upgrade for maximum consistency. To maximize signal stability, our center routinely performs rigorous daily quality control. We conduct a standardized quality assurance framework through the use of a standard system structural and functional phantom to ensure that quantitative measures including signal-to-noise ratio (SNR), system drift, and image uniformity derived from MR data are stable and comparable over time.

The quantitative group-level analysis revealed that the test-retest reliability values of FC (both seed-based and FC matrix) were moderate for subject-wise CCC and fair for voxel/FC-wise ICC while the BOLD variability was good for subject-wise CCC and moderate for ROI-wise ICC (see Supplementary Table 1), which is comparable with previous studies (7-9). Our findings suggest that functional connectivity strength and BOLD time series fluctuations are comparable between the two scanners. Moreover, in the current study, we have kept the scanner type consistent for both time points within each individual. We have also added the scanner type variable as covariates in all statistical analyses.

**The in-scanner motion of the participants did not reflect the BCI-intervention effect**

There was no time (pre- and post-) and group (ADHD-NI and ADHD-I groups) interaction on FD and DVAS for both the complete and sub-cohort sample (see Supplementary Table 4). This was true for both rs-fMRI data with and without motion scrubbing. Moreover, it did not reveal any differences of FD and DVAS between the two time-points within each group. Our results suggested that in our sample, the in-scanner head motion parameters were comparable across the two time-points and did not reflect BCI-based intervention effect, which mitigate the concern that the observed BCI-based functional network changes is confounded by motion differences. In addition, we have performed motion scrubbing and controlled for the individual effect of number of frames in the statistical analysis of the current study (see Supplementary Table 3).

**References**

1. Bullmore E, Sporns O. The economy of brain network organization. Nature Reviews Neuroscience. 2012;13(5):336-49.

2. Rubinov M, Sporns O. Complex network measures of brain connectivity: uses and interpretations. Neuroimage. 2010;52(3):1059-69.

3. Chong JSX, Liu S, Loke YM, Hilal S, Ikram MK, Xu X, et al. Influence of cerebrovascular disease on brain networks in prodromal and clinical Alzheimer’s disease. Brain. 2017;140(11):3012-22.

4. Seeley WW, Crawford RK, Zhou J, Miller BL, Greicius MD. Neurodegenerative diseases target large-scale human brain networks. Neuron. 2009;62(1):42-52.

5. Chen G, Taylor PA, Haller SP, Kircanski K, Stoddard J, Pine DS, et al. Intraclass correlation: improved modeling approaches and applications for neuroimaging. Human brain mapping. 2018;39(3):1187-206.

6. Lawrence I, Lin K. A concordance correlation coefficient to evaluate reproducibility. Biometrics. 1989:255-68.

7. Guo CC, Kurth F, Zhou J, Mayer EA, Eickhoff SB, Kramer JH, et al. One-year test–retest reliability of intrinsic connectivity network fMRI in older adults. Neuroimage. 2012;61(4):1471-83.

8. Wang J, Ren Y, Hu X, Nguyen VT, Guo L, Han J, et al. Test–retest reliability of functional connectivity networks during naturalistic fMRI paradigms. Human brain mapping. 2017;38(4):2226-41.

9. Song J, Desphande AS, Meier TB, Tudorascu DL, Vergun S, Nair VA, et al. Age-related differences in test-retest reliability in resting-state brain functional connectivity. PLoS One. 2012;7(12):e49847.

**Supplementary Table 1: Test-retest reliability of functional connectivity (FC) measures and the variability of BOLD time series**

|  | Seed-based FC map | 141-ROI-based FC matrix | Variability of BOLD time series |
| --- | --- | --- | --- |
| subject-wise CCC (mean ± SD) | 0.55 ± 0.11 | 0.53 ± 0.16 | 0.64 ± 0.17 |
| voxel/FC-wise ICC (mean ± SD) | 0.34 ± 0.17 | 0.28 ± 0.15 | 0.40 ± 0.21 |

**Supplementary Table 2: Differential changes in intra- and inter-network functional connectivity of the brain networks with and without BCI-based intervention.**

|  | Group and time interaction effect | ADHD-NI group  Pre < Post | ADHD-I group  Pre > Post |
| --- | --- | --- | --- |
| SalVenAttn- SalVenAttn | 0.019^*^ | 0.11 | 0.21 |
| SalVenAttn-DorAttn | 0.035^*^ | 0.075 | 0.23 |
| SalVenAttn-SomMot | 0.014^*^ | 0.053 | 0.21 |
| SalVenAttn-subcortical | 0.050^*^ | 0.079 | 0.17 |
| SomMot-Cont | 0.049^*^ | 0.077 | 0.23 |

Note: Values in each cell represent the p-values. The first column indicated significant group and time interaction effect (* represents the threshold of p < 0.05) and the second and third columns represent results from paired t-tests. Abbreviations: SalVenAttn: Salience/Ventral attention network; DorAttn: Dorsal attention network; SomMot: somatomotor network; Cont: Executive control network.

**Supplementary Table 3: Differential changes in topological measures of brain functional networks with and without BCI-based intervention.**

|  | | Group and time interaction effect | ADHD-NI group  Pre < Post | ADHD-I group  Pre > Post |
| --- | --- | --- | --- | --- |
| Degree | LH-ContA-PFCl | 0.0052^*^ | 0.090 | 0.011 |
|  | LH-ContB-PFCl | 0.00060^*^ | 0.036 | 0.014 |
|  | LH-SalVentAttnB-PFCmp | 0.0060^*^ | 0.32 | 0.0012^*^ |
|  | RH-SalVentAttnA-PrCv | 0.0014^*^ | 0.034 | 0.099 |
|  | RH-SalVentAttnB-PFCmp | 0.0066^*^ | 0.28 | 0.0060^*^ |
|  | | Group and time interaction effect | ADHD-NI group  Pre > Post | ADHD-I group  Pre < Post |
| Closeness | LH-ContB-PFCl | 0.0024^*^ | 0.021 | 0.10 |
|  | | Group and time interaction effect | ADHD-NI group  Pre < Post | ADHD-I group  Pre > Post |
| Clustering coefficient | RH-DefaultB-PFCv | 0.0080^*^ | 0.068 | 0.18 |
|  | LH-SalVentAttnA-FrMed | 0.0052^*^ | 0.0016^*^ | 0.088 |

Note: Values in each cell represent the p-values. The first column indicated significant group and time interaction effect and the second and third columns represent results from paired t-tests (* represents the threshold of p < 0.01). Abbreviations: ContA/B: Executive control network A/B (A or B refers to the subnetworks); SalVenAttn: Salience/Ventral attention network; DorAttn: Dorsal attention network; Default: Default mode network; PrCv: precentral ventral frontal cortex; PFCmp: medial posterior prefrontal cortex; PFCl: lateral prefrontal cortex; SPL: superior parietal lobule; FrMed: medial frontal cortex; PFCv: ventral prefrontal cortex.

**Supplementary Table 4: Changes in motion parameters (FD and DVARS) before and after BCI-based intervention.**

|  | | FD | | | DVARS | | |
| --- | --- | --- | --- | --- | --- | --- | --- |
|  |  | Group and time interaction effect | Time effect of ADHD-I | Time effect of ADHD-NI | Group and time interaction effect | Time effect of ADHD-I | Time effect of ADHD-NI |
| ADHD-I, N=33; ADHD-NI, N=18 | Data without motion scrubbing | 0.46 | 0.28 | 0.99 | 0.47 | 0.41 | 0.89 |
|  | Data with motion scrubbing | 0.37 | 0.64 | 0.60 | 0.53 | 0.46 | 0.92 |
| ADHD-I, N=18; ADHD-NI, N=11 | Data without motion scrubbing | 0.13 | 0.73 | 0.30 | 0.27 | 0.67 | 0.34 |
|  | Data with motion scrubbing | 0.68 | 0.79 | 0.58 | 0.32 | 0.81 | 0.46 |

Note: Values in each cell represented the p-values (two-tailed). There were no time differences in FD and DVARS within each group, no group and time interactions, and no differences between groups at each time point (two-sample t-test) (p > 0.05).

**SUPPLEMENTARY FIGURES**

**

**

**Supplementary Figure 1. BCI intervention in ADHD renormalized the brain functional network topology.** A. Nodes showing significant time and group interaction effect on nodal degree, clustering coefficient or closeness are presented. Brain network topology exhibited significant group and time interaction in nodal degree (B), clustering coefficient (C), and closeness (D) (p < 0.05). Error bars represent standard errors. Abbreviations: ContA/B, Executive control network A/B (A and B refers to the subnetworks); SomMot, somatomotor network, VisPeri, Peripheral visual; VisCent, Central visual; SalVenAttn, Salience/Ventral attention network; DorAttn, Dorsal attention network; Default, Default mode network; PFCl, lateral prefrontal cortex; PFCd, dorsal prefrontal cortex; SPL, superior parietal lobule; Cingp, posterior cingulate sulcus; Aud, auditory cortex; Striate, striate cortex; PFCm, medial prefrontal cortex; ExStr, extrastriate; ExStrInf, inferior extrastriate cortex; FrMed, medial frontal cortex; ParMed, medial parietal cortex; PFCmp, medial posterior prefrontal cortex; PrCv, precentral ventral frontal cortex; Ins, insula cortex; PFCv, ventral prefrontal cortex; PrC, precentral gyrus; IPL, inferior parietal lobule; ParOper, parietal operculum; Cinga, anterior cingulate sulcus; S2, secondary somatosensory cortex; Temp, temporal cortex.

**

**

**Supplementary Figure 2. Changes of nodal graph metrics by BCI intervention in ADHD is associated with behavioral improvement.** Less decrease of the closeness resulted in more behavior improvement of the internalizing problems in children with ADHD (A, B & C). Less increase of the clustering coefficient resulted in more behavior improvement of the internalizing problems in children with ADHD (D & E). Less increase of the clustering coefficient resulted in more behavior improvement of the inattention in children with ADHD (F & G). Abbreviations: VisCent, Central visual; SalVenAttn, Salience/Ventral attention network; FrMed, medial frontal cortex; PrCv, precentral ventral frontal cortex; PFCv, ventral prefrontal cortex; PrC, precentral gyrus; ParOper, parietal operculum; ExStr, extrastriate.
